# Early treatment with isoflurane improves cardiac function and increases survival in a rat model of TAKO-TSUBO cardiomyopathy

**DOI:** 10.1186/2197-425X-3-S1-A800

**Published:** 2015-10-01

**Authors:** J Oras, B Redfors, E Omerovic, S Ricksten, H Seemann-Lodding

**Affiliations:** Sahlgrenska University Hospital, Anesthesiology and Intensive Care Medicine, Gothenburg, Sweden; Sahlgrenska University Hospital, Wallenberg Laboratory, Gothenburg, Sweden

## Introduction

Tako-tsubo cardiomyopathy (TCM) is an acute cardiac syndrome with severe hypokinesia of the left ventricle (LV), often affecting the apex causing apical ballooning and heart failure. Sympathetic stress is a trigger of TCM and is probably underdiagnosed and not uncommon in ICU patients [1]. We have developed an experimental rat model of TCM, in which LV apical akinesia develops after intraperitoneal (i.p.) injection of isoprenaline with many similarities to clinical TCM [2]. We recently demonstrated that pre-treatment with isoflurane could **prevent** the development of LV apical akinesia in this TCM animal model [3].

## Objectives

In the present study, we examined whether isoflurane, when used for **treatment**, could attenuate the degree of LV dysfunction and reduce mortality in a TCM animal model.

## Methods

TCM was induced in seventy-five propofol sedated animals by intraperitonial injection of isoprenaline (50 mg/kg). Within 90 minutes after injection, the animals develop tako-tsubo-like heart failure with apical dyskinesia/akinesia and reduced cardiac output [2]. The animals were randomized to either inhalation with no isoflurane (CONTROL, n=15) or inhalation with 1% isoflurane before (ISOFL 0, n=15), 10 min (ISOFL 10, n=15), 30 min (ISOFL 30, n=15) and 120 min (ISOFL 120, n=15) after induction of TCM. Evaluation of cardiac function with echocardiography was performed 90 minutes after isoprenaline in all animals. Extent of apical akinesia was expressed as percentage of LV endocardial length that was akinetic. After the experimental procedure, the animals were monitored for 48 hours for assessment of survival.

## Results

**Figure 1:**
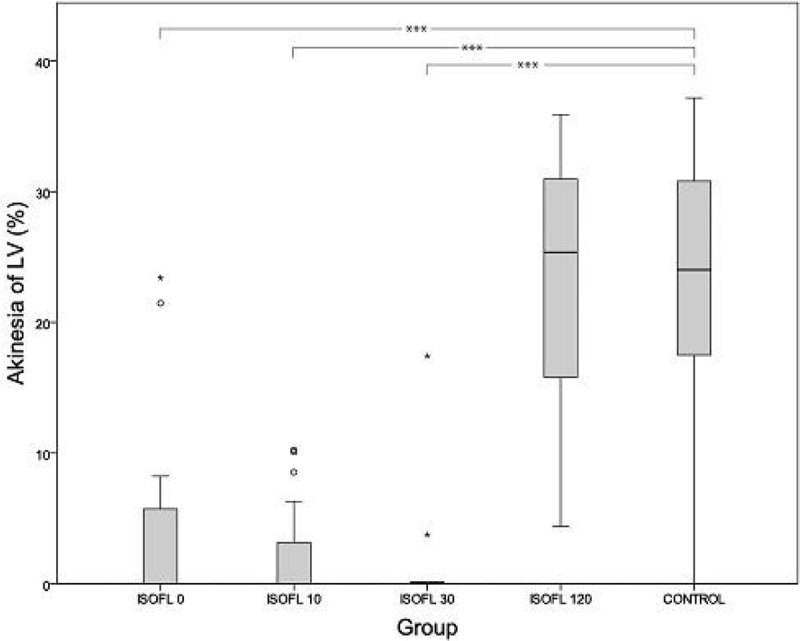
Figure 1

**Figure 2:**
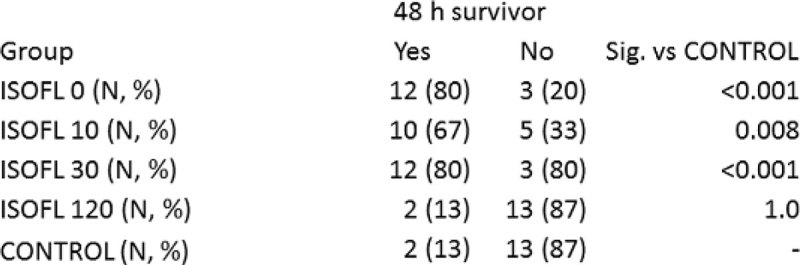
Figure 2

Early institution of treatment with isoflurane, attenuated the degree of LV dysfunction after isoprenaline injection, as the degree of akinesia was lower in ISOFL 0, ISOFL 10, ISOFL 30 compared to CONTROL (Figure 1). 48 hours survival was higher in ISOFL 0, ISOFL 10, ISOFL 30 versus CONTROL, while there was no difference in 48 hours survival between ISOFL 120 and CONTROL (Figure 2). In a multivariable model, isoflurane inhalation within 30 minutes (ISOFL 0, ISOFL 10, ISOFL 30), was an independent predictor of 48 hour survival.

## Conclusions

In this animal model of TCM, isoflurane treatment, when started early after induction of TCM, highly attenuated degree of LV apical akinesia. This was accompanied by a clearly improvement of survival. Isoflurane sedation in the ICU could be a beneficial strategy in patients suffering from hyper-adrenergic conditions.
